# Paediatric acute lymphoblastic leukaemia and caesarean section: A report from the United Kingdom Childhood Cancer Study (UKCCS)

**DOI:** 10.1111/ppe.12662

**Published:** 2020-04-29

**Authors:** Audrey Bonaventure, Jill Simpson, Pat Ansell, Eve Roman

**Affiliations:** ^1^ Epidemiology and Cancer Statistics Group Department of Health Sciences University of York York UK; ^2^ CRESS Université de Paris INSERM UMR 1153 Epidemiology of Childhood and Adolescent Cancers Team Villejuif France

**Keywords:** caesarean, childhood leukaemia, delivery, epidemiology

## Abstract

**Background:**

Reports have suggested that children born by caesarean initiated before labour onset may be at increased risk of developing acute lymphoblastic leukaemia (ALL). However, with most data being derived from case‐control study interviews, information on the underpinning reasons for caesarean section is sparse, and evidence is conflicting.

**Objectives:**

Use clinical records compiled at the time of delivery to investigate the association between childhood ALL and caesarean delivery; examining timing in relation to labour onset, and reasons for the procedure.

**Methods:**

Data are from the UK Childhood Cancer Study, a population‐based case‐control study conducted in the 1990s, when caesarean section rates were relatively low, in England, Scotland, and Wales. Children with ALL were individually matched to two controls on sex, date of birth, and region of residence. Information on mode of delivery and complications was abstracted from obstetric records. Odds ratios (OR) and 95% confidence intervals (CI) were calculated using logistic regression models adjusted for matching variables and relevant covariates.

**Results:**

Around 75% of the 1034 cases and 1914 controls were born through unassisted vaginal delivery. Caesarean delivery was as frequent in cases and controls (OR 1.07, 95% CI 0.84, 1.36). No association was observed between ALL and caesarean delivery either during or before labour, with adjusted ORs of 1.08 (95% CI 0.78, 1.48) and 1.09 (95% CI 0.78, 1.53), respectively. For B‐cell ALL, the ORs were 1.14 (95% CI 0.81, 1.59) for caesarean during labour and 1.21 (95% CI 0.85, 1.72) for prelabour. The underpinning reasons for caesarean delivery differed between cases and controls; with preeclampsia, although very rare, being more common amongst cases born by caesarean (OR 8.91, 95% CI 1.48, 53.42).

**Conclusions:**

Our obstetric record‐based study found no significant evidence that caesarean delivery increased the risk of childhood ALL, either overall or when carried out before labour.

**Synopsis**

1 **Study question**

Does birth by caesarean delivery increase the risk of childhood leukaemia?

2 **What is already known**

Previous reports are conflicting, but some have suggested an increased risk of childhood leukaemia following caesarean delivery initiated before labour onset.

3 **What this study adds**

Obstetric records were used to look at type of delivery, time of labour onset, and reason for caesarean delivery. No strong evidence of an association between leukaemia and caesarean delivery was found, either generally or before labour onset. However, the reasons for the caesarean differed between children with and without leukaemia; preeclampsia occurring more frequently in mothers of affected children.

## 1 BACKGROUND

Acute lymphoblastic leukaemia (ALL) is the commonest paediatric malignancy, representing around a third of cancers diagnosed in children (0‐14 years) in high‐income countries. The characteristic incidence peak between 2 and 5 years of age^1^ has provided the foundation for several aetiological hypotheses, most notably, concerning a potential role of exogenous factors on the emergent immune system and subsequent risk of ALL.^2^

In this context, it has been suggested that because children born by caesarean delivery may not be subject to the same hormonal and microbial challenges as those born vaginally, their immune development could be altered, which in turn could increase their risk of ALL. Although findings from the majority of studies examining the relationship with caesarean delivery have provided little support for this hypothesis,^3^, ^4^, ^5^, ^6^, ^7^, ^8^, ^9^ some investigators observed that children delivered by caesarean,^10^ especially if performed before the onset of labour,^11^, ^12^ may be at increased ALL risk. A further case‐control study, finding no overall association with caesarean delivery, either before or during labour, reported an increased risk with prelabour caesarean among children diagnosed with ALL before three years of age^13^; and a Californian birth record linkage study reported a 20% increased risk of ALL with caesarean delivery (pre‐ and post‐labour combined) in children aged 2‐4 years.^11^

The Childhood Leukaemia International Consortium (CLIC) pooled analysis used algorithms to classify the, mostly self‐reported, mode of delivery across the 13 included studies.^14^ Four of these asked mothers about the reason for the caesarean; categorizing caesareans as “prelabour” if the reason given was either previous caesarean or multiple birth.^14^ Likewise, the Californian record linkage study used “elective” as a marker for prelabour.^11^

With a view to investigating the association between caesarean section and ALL in more depth, this report presents findings from a detailed examination of obstetric records collected during the United Kingdom Childhood Cancer Study (UKCCS).^15^ Conducted in the early 1990s, the median year of birth was 1989, a time when caesareans represented only 12% of deliveries in the UK,^16^ less than half the current level.^17^, ^18^

## 2 METHODS

Data are from a population‐based case‐control study specifically designed to examine the potential aetiological role of a range of potential risk factors, including perinatal and reproductive events (UKCCS).^15^, ^19^ This report used obstetric records of 1034 mothers of children diagnosed with ALL (cases) in England and 1914 mothers of children without cancer (controls); each case being individually matched to at least one control on sex, date of birth, and region of residence. Data relating to mode of delivery, timing (before or during labour), and reasons for caesarean delivery were extracted directly from obstetric records. Detailed information about abstraction methods is presented elsewhere.^15^

Caesarean delivery was classified as prelabour if the medical record stated that labour had not started before it was performed. Odds ratios (OR) and 95% confidence intervals (CI) were estimated using unconditional logistic regression, with initial adjustment for matching variables. Additional adjustment for deprivation index quintile, birthweight (≤2499, 2500‐3999, ≥4000 g), birth order (first‐born, higher order), and maternal age (<26, 26‐35, >35 years) was also performed. Age at diagnosis and hyperdiploidy status were also examined. Analyses were conducted using Stata 15.1 (StataCorp 2017).

## 3 RESULTS

Characteristics of cases and controls are presented in Table 1. As expected, children who developed ALL were, on average, slightly heavier at birth than controls, mostly due to B‐cell ALL (adjusted OR _4000 g or more_ 1.32, 95% CI 1.02, 1.71). Children with B‐ALL tended to be first‐born more often than controls (OR 1.17, 95% CI 0.99, 1.38). No differences between cases and controls were evident for gestational age or area‐based deprivation.

**Table 1:** Numbers of cases and controls, odds ratios (OR), and 95% confidence interval (CI), distributed by baseline and delivery characteristics: UKCCS, acute lymphoblastic leukaemia

		Controls	Acute lymphoblastic leukaemia (ALL)					
			Total			B‐ ALL		
		N (%)	N (%)	OR (95%CI) a	OR (95% CI) fully adjusted b	N (%)	OR (95%CI) a	OR (95% CI) fully adjusted b
Total		1914 (100)	1034 (100)			827 (100)		
Age at diagnosis (years)	Median	4.1	4.3			4.0		
Year of birth	Median	1989	1989			1989		
Sex (male)	Male	1068 (55.8)	578 (55.9)			451 (54.5)		
Birthweight (g)	≤2499	124 (6.5)	48 (4.6)	0.71 (0.51, 1.00)		40 (4.8)	0.75 (0.52, 1.09)	
	2500‐3999	1602 (83.7)	870 (84.1)	1.00 (Reference)		683 (82.6)	1.00 (Reference)	
	≥4000	188 (9.8)	116 (11.2)	1.14 (0.89, 1.46)		104 (12.6)	1.32 (1.02, 1.71)	
Gestational age (weeks)^c^	<37	123 (6.5)	60 (5.9)	0.91 (0.66, 1.26)		45 (5.5)	0.85 (0.60, 1.21)	
	37‐40	1371 (72.0)	726 (70.8)	1.00 (Reference)		589 (71.8)	1.00 (Reference)	
	>40	411 (21.6)	240 (23.4)	1.10 (0.92, 1.33)		186 (22.7)	1.05 (0.86, 1.28)	
Birth order	1	826 (43.2)	461 (44.6)	1.06 (0.91‐1.23)		388 (46.9)	1.17 (0.99‐1.38)	
	>1	1088 (56.8)	573 (55.4)	1.00 (Reference)		439 (53.1)	1.00 (Reference)	
Deprivation (quintiles)	Least deprived	397 (20.7)	212 (20.5)	1.00 (Reference)		172 (20.8)	1.00 (Reference)	
		394 (20.6)	214 (20.7)	1.01 (0.80, 1.28)		169 (20.4)	1.00 (0.77, 1.29)	
		414 (21.6)	202 (19.5)	0.91 (0.72, 1.15)		169 (20.4)	0.94 (0.73, 1.21)	
		361 (18.9)	205 (19.8)	1.07 (0.84, 1.36)		156 (18.9)	1.00 (0.77, 1.29)	
	Most deprived	348 (18.2)	201 (19.4)	1.09 (0.85, 1.40)		161 (19.5)	1.07 (0.82, 1.40)	
Mode of delivery								
Unassisted vaginal		1441 (75.3)	778 (75.2)	1.00 (Reference)	1.00 (Reference)	613 (74.1)	1.00 (Reference)	1.00 (Reference)
Assisted vaginal		239 (12.5)	128 (12.4)	1.00 (0.79, 1.26)	0.98 (0.76, 1.25)	104 (12.6)	1.01 (0.79, 1.30)	0.95 (0.73, 1.23)
Caesarean		234 (12.2)	128 (12.4)	1.01 (0.80, 1.28)	1.07 (0.84, 1.36)	110 (13.3)	1.10 (0.86, 1.41)	1.15 (0.89, 1.48)
After labour onset^d^		118 (6.2)	67 (6.5)	1.06 (0.77, 1.44)	1.08 (0.78, 1.48)	58 (7.0)	1.15 (0.82, 1.59)	1.14 (0.81, 1.59)
Before labour onset^d^		111 (5.8)	60 (5.8)	1.00 (0.72, 1.39)	1.09 (0.78, 1.53)	51 (6.2)	1.08 (0.77, 1.53)	1.21 (0.85, 1.72)
	Planned	85 (4.4)	46 (4.5)	1.00 (0.69, 1.45)	1.07 (0.74, 1.56)	39 (4.7)	1.07 (0.72, 1.59)	1.19 (0.80, 1.76)
	Emergency	26 (1.4)	14 (1.4)	0.99 (0.51, 1.91)	1.16 (0.59, 2.29)	12 (1.5)	1.12 (0.56, 2.24)	1.27 (0.62, 2.59)

a Models adjusted for matching variables: sex, date of birth, study region.

b Models adjusted for matching variables and deprivation quintile, birthweight category, birth order category, and maternal age category.

c Missing from 9 controls and 8 cases.

d Missing caesarean timing data for 5 controls and 1 case.

Around 88% of cases and controls were delivered vaginally and 12% by caesarean (Table 1). No significant associations between prelabour caesarean delivery and childhood ALL were noted, with ORs of 1.09 (95% CI 0.78, 1.53) for any prelabour caesarean; 1.07 (95% CI 0.74‐1.56) for planned prelabour, and 1.16 (95% CI 0.59‐2.29) for emergency prelabour.

For B‐cell ALL, the ORs adjusted for the matching variables were 1.15 (0.82‐1.59) for caesarean performed during labour, and 1.08 (0.77‐1.53) prelabour. Only birthweight slightly increased these estimates on adjustment. Fully adjusted B‐ALL ORs for caesarean were 1.14 (95% CI 0.81, 1.59) during labour and 1.21 (95% CI 0.85, 1.72) prelabour; ORs for planned prelabour and emergency prelabour being 1.19 (95% CI 0.80, 1.76) and 1.27 (95% CI 0.62, 2.59), respectively. No age‐related associations were detected for ALL and prelabour caesarean: OR_0‐4 years_: 0.92, 95% CI 0.60, 1.40; OR_5‐9 years_: 1.10, 95% CI 0.58, 2.11; OR_10‐14 years:_ 1.26, 95% CI 0.52, 3.07. Furthermore, analyses by hyperdiploidy status provided no support for an association between ALL (or B‐ALL) and prelabour caesarean delivery (OR_hyperdiploid_ 0.97, 95% CI 0.57, 1.65).

Although most prelabour caesarean deliveries were elective/planned, around 23% were emergencies, in both cases (14/60) and controls (26/111). Table 2 shows the main reason underpinning caesarean delivery. Foetal distress predominates during labour (58.5% in controls, 53.7% in cases).By contrast, prior caesarean (often elective/planned) was the main reason for prelabour caesareans (49.6% of controls, 35.0% of cases), with multiplicity accounting ~4%‐5% of the total. Nonetheless, around half of prelabour caesareans occurred for other reasons: breech/unstable lie and placenta praevia combined recorded in around 25%. Often performed prelabour, caesarean deliveries for preeclampsia were more common among cases than controls (OR 8.91, 95% CI 1.48, 53.42; based on 6 cases and 2 controls).

**Table 2:** Main reasons for caesarean delivery recorded in obstetrical records, ordered according to the overall frequency among controls

	All caesareans		During labour		Prelabour					
			Emergency		Total		Elective/planned		Emergency	
	Controls N (%)	Cases N (%)	Controls N (%)	Cases N (%)	Controls N (%)	Cases N (%)	Controls N (%)	Cases N (%)	Controls N (%)	Cases N (%)
Total^a^	229 (100)	127 (100)	118 (100)	67 (100)	111 (100)	60 (100)	85 (100)	46 (100)	26 (100)	14 (100)
Foetal distress^b^	78 (34.1)	41 (32.3)	69 (58.5)	36 (53.7)	9 (8.1)	5 (8.3)	1 (1.2)	1 (2.2)	8 (30.8)	4 (28.6)
Prior caesarean	67 (29.3)	24 (18.9)	12 (10.2)	3 (4.5)	55 (49.6)	21 (35.0)	52 (61.2)	19 (41.3)	3 (11.5)	2 (14.3)
Breech/unstable lie^c^	35 (15.3)	26 (20.5)	19 (16.1)	15 (22.4)	16 (14.4)	11 (18.3)	15 (17.7)	8 (17.4)	1 (3.9)	3 (21.4)
Obstructive factors/macrosomia/failure to progress	12 (5.2)	10 (7.9)	10 (8.5)	10 (14.9)	2 (1.8)	—	2 (2.4)	—	—	—
Placenta praevia/abruption	12 (5.2)	3 (2.4)	—	—	12 (10.8)	3 (5.0)	3 (3.5)	3 (6.5)	9 (34.6)	—
Failed induction	6 (2.6)	—	3 (2.5)	—	3 (2.7)	—	—	—	3 (11.5)	—
Multiplicity	5 (2.2)	4 (3.2)	1 (0.9)	1 (1.5)	4 (3.6)	3 (5.0)	4 (4.7)	2 (4.4)	—	1 (7.1)
Preeclampsia	2 (0.9)	7 (5.5)	—	1 (1.5)	2 (1.8)	6 (10.0)	1 (1.2)	4 (8.7)	1 (3.9)	2 (14.3)
Other	12 (5.2)	12 (9.4)	4 (3.4)	1 (1.5)	8 (7.2)	11 (18.3)	7 (8.2)	9 (19.6)	1 (3.9)	2 (14.3)

a 1 case and 5 controls without times of caesarean are excluded.

b Includes heart, meconium, and reduced foetal movement.

c Includes mal/cord presentation and high head.

## 4 COMMENT

Based on reliable clinical data collected at the time of birth, our findings do not provide strong support for the previously reported association between caesarean delivery and childhood ALL. No evidence of an effect on childhood ALL was observed either overall, before or during labour, or between planned and emergencies caesarean deliveries. Furthermore, the weak positive association between caesarean and B‐ALL was not specific to those conducted prelabour. No association specific to hyperdiploid ALL, or to any age group, was noted.

In our study, 12% of cases and controls were delivered by caesarean. This comparatively low proportion is consistent with contemporaneous national data,^16^ and with the range observed in previous childhood cancer case‐control studies (7%–38% as summarized by Marcotte and colleagues^14^). Lying within the WHO ideal range of 10%‐15%,^17^ our findings provide an important benchmark predating the dramatic rise in caesarean deliveries observed in many countries.^17^, ^18^

The pooled CLIC analysis reported a 23% increased risk of ALL in children born by prelabour caesarean, these findings being based on a definition that did not account for all indications.^14^ Whilst our findings for B‐ALL are broadly compatible with such an association, no evidence for a prelabour‐specific or age‐specific effect was observed.^11^, ^13^ More recently, another study reported a 2.67 (95% CI 1.09, 6.57) increased risk of ALL in children born by caesarean delivery, but the findings are difficult to interpret due to data exclusions (eg multiple pregnancies, delivery before 37 weeks, emergency deliveries).^20^ Conversely, a nationwide record linkage study, using data from birth and cancer registries including 7 029 843 children from Denmark, Finland, and Sweden, found no association between childhood leukaemia and caesarean section, either elective or emergency (adjusted HR 1.02, 95% CI 0.92, 1.13).^9^

It has been hypothesized that a positive association between prelabour caesarean and childhood ALL could be related to a lack of exposure to vaginal microbiota^14^; and other known caesarean‐related hormonal and epigenetic mechanisms could also be implicated.^21^ In order to properly investigate the relation between caesarean and childhood ALL and eliminate confounding, it is crucial to also account for the reason for the caesarean delivery. For instance, one could hypothesize that neonates who develop ALL in utero may suffer foetal distress in late pregnancy, therefore prompting an emergency caesarean; the possibility of indication bias warrants more studies with relevant information from medical records. Moreover, one cannot yet rule out confounding by indication on maternal health‐related events. While based on small numbers, our results suggest that preeclampsia could be such a confounder. Birth order, being related to caesarean indication, preeclampsia and childhood ALL, also appears as a likely confounder, as does macrosomia, which used to be an indication for caesarean delivery.^22^ Interestingly, in our study, adjusting for birth weight was the only factor to increase the point estimates.Whether caesarean could be causally related to childhood ALL, or whether they could share a common cause, also needs to be addressed.

Caesarean deliveries have become increasingly common, in some countries worryingly so.^17^ Clearly studying the potential adverse effects that caesareans could have on subsequent child (and maternal) health is complex, and needs to look in detail at the underpinning reasons for the procedures. In order to gain insight into potential mechanisms, future studies will need to access comprehensive clinical data with information on delivery mode and indication for caesarean.

## CONFLICT OF INTEREST

None declared.
